# T1 mapping for myocardial extracellular volume measurement by cardiovascular magnetic resonance: bolus only vs primed infusion technique

**DOI:** 10.1186/1532-429X-15-S1-O14

**Published:** 2013-01-30

**Authors:** Steven K White, Daniel Sado, Marianna Fontana, Sanjay M Banypersad, Viviana Maestrini, Stefan K Piechnik, Matthew D Robson, Derek J Hausenloy, Amir M Sheikh, Philip N Hawkins, James Moon

**Affiliations:** 1 The Heart Hospital Imaging Centre, The Heart Hospital, London, UK; 2 Cardiothoracic Surgery, The Heart Hospital, London, UK; 3 The Hatter Cardiovascular Institute, University College London, London, UK; 4 National Amyloidosis Centre, University College London, London, UK; 5 OCMR, Oxford University, Oxford, UK

## Background

Myocardial ECV can be measured using T1 mapping before and after contrast if the contrast agent distribution between blood:myocardial is at equilibrium. Equilibrium distribution can be achieved with a primed contrast infusion (EQ-CMR), or may be approximated by the dynamic equilibration achieved by delayed post bolus measurement. This bolus only approach is highly attractive but currently limited data support its use. We compared the bolus only technique with two gold standards: collagen volume fraction (CVF) from myocardial biopsy in aortic stenosis (AS), and the infusion technique in five representative conditions.

## Methods

147 subjects were studied: healthy volunteers (n=50); hypertrophic cardiomyopathy (HCM, n=25); severe AS (n=22); amyloid (n=20); and chronic myocardial infarction (n=30). Bolus only (at 15 minutes) and infusion ECV measurements were performed and compared. In 18 subjects with severe AS the results were compared to histological CVF.

## Results

ECV by bolus only technique correlated well with CVF (n=18, r2=0.56, p=<0.01) but the r2 was higher with the infusion technique (r2=0.71, p=<0.01). Across health and disease, there was high correlation between the techniques (r2=0.97). However, in diseases of high ECV (amyloid, HCM LGE, and infarction), Bland-Altman analysis indicates the bolus only technique consistently and increasingly overestimates ECV.

## Conclusions

Bolus only, T1 mapping derived ECV measurement is sufficient for ECV measurement across a range of cardiac diseases and this approach is histologically validated in AS. However, when ECV is above 0.4, there is overestimation compared to the infusion approach.

## Funding

British Heart Foundation.

**Figure 1:**
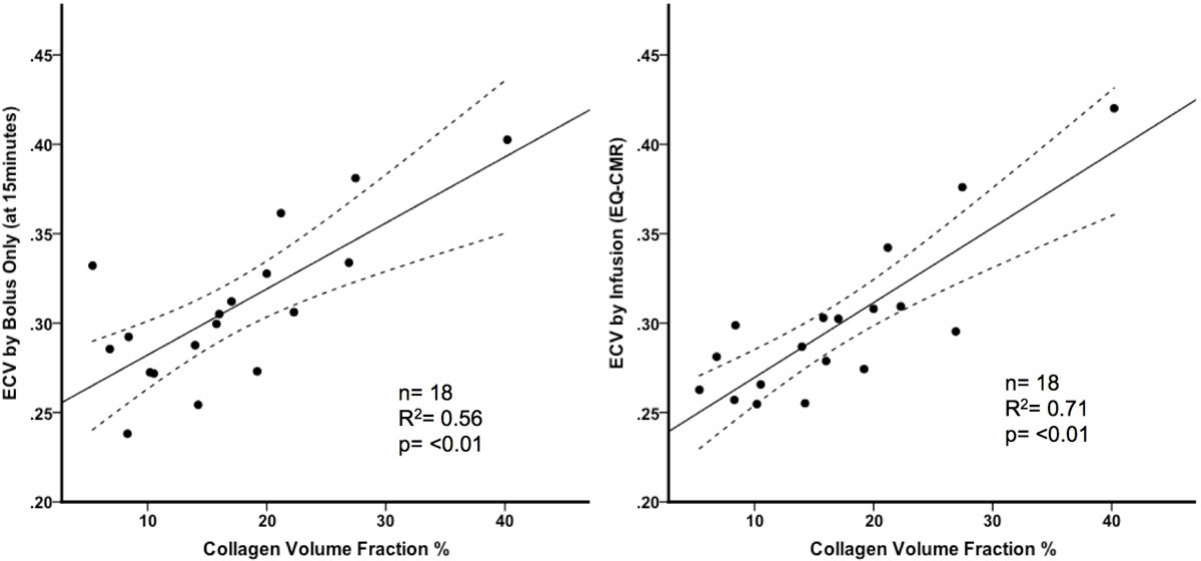
Correlation with histological collagen volume fraction in severe aortic stenosis. Both the bolus only technique (at 15 minutes) and the infusion techniques correlate well with CVF but the r2 value is higher with the infusion technique (0.56 vs 0.71).

**Figure 2:**
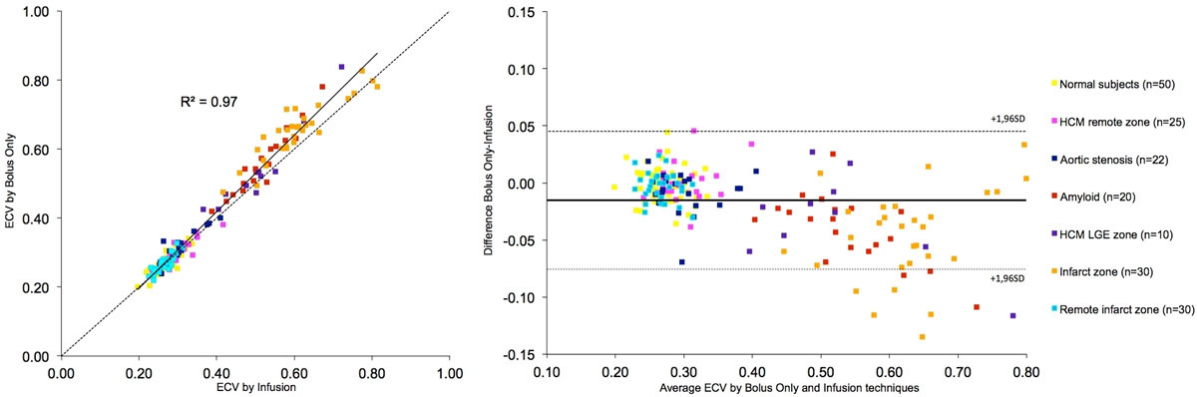
Correlation and Bland-Altman Analysis of the comparison between Bolus only and Infusion techniques across health and disease. There is a bias towards ECV overestimation in those diseases where the ECV >0.4, and this appears to increase with increasing ECV (amyloid, HCM LGE, chronic infarct zones). The dashed lines denote the line of identity in the scatterplot, and the mean ± 1.96SD in the Bland-Altman plots for all subjects combined.

